# Consciousness and the Invention of Morel

**DOI:** 10.3389/fnhum.2013.00061

**Published:** 2013-03-05

**Authors:** Lampros Perogamvros

**Affiliations:** ^1^Department of Psychiatry, Division of Neuropsychiatry, University Hospitals of GenevaGeneva, Switzerland; ^2^Department of Neuroscience, University of GenevaGeneva, Switzerland

**Keywords:** consciousness, virtual reality, brain reading, Adolfo Bioy Casares, Morel, dreaming

## Abstract

A scientific study of consciousness should take into consideration both objective and subjective measures of conscious experiences. To this date, very few studies have tried to integrate *third-person data*, or data about the neurophysiological correlates of conscious states, with *first-person data*, or data about subjective experience. Inspired by Morel's invention (Casares, 1940), a literary machine capable of reproducing sensory-dependent external reality, this article suggests that combination of virtual reality techniques and brain reading technologies, that is, decoding of conscious states by brain activity alone, can offer this integration. It is also proposed that the multimodal, simulating, and integrative capacities of the dreaming brain render it an “endogenous” Morel's machine, which can potentially be used in studying consciousness, but not always in a reliable way. Both the literary machine and dreaming could contribute to a better understanding of conscious states.

## Introduction

### The science of subjective consciousness

Conscious states are inner states and processes of awareness, with undeniable neurobiological underpinnings (Searle, 2000). These states are by definition subjective; every scientific investigation of consciousness should deal with this subjectivity (Searle, 2000; Chalmers, 2004). In addition, most theories of consciousness assert that all conscious experiences have specific qualitative attributes that differentiate them from each other (qualitativeness), and a unified nature (unity), which cannot be reduced to independent components (Tononi, 2008).

It has been proposed that a science of consciousness should systematically integrate *third-person data*, or data about the neurophysiological correlates of conscious states, with *first-person data*, or data about the distinctive qualities of subjective experience (Chalmers, 2004). Indeed, neurophysiology alone is not sufficient to describe a conscious state without taking into account the first-person's point of view, and vice versa. Very few studies have tried to integrate both kinds of data together (Lutz et al., 2002; Dehaene et al., 2003). Moreover, while great progress has been done regarding our methods for gathering third-person data (e.g., neuroimaging methods or single-cell recording with electrode implantation), to this date there is no sufficient scientific description of subjective conscious experience, apart from verbal report. However, verbal report represents for the scientist only an indirect observation of a person's subjective experiences and is prone to certain limitations: language may misdescribe or may be unable to describe a subjective experience or the person may voluntarily hide or lie about his/her experience (Hospers, 1997, p. 93).

Therefore, the relatively new science of subjective consciousness is in urgent need of novel methods for gathering first-person data and, in parallel, of ways to integrate this data with their neurophysiological correlates. Here, we will see that such an integrative model of consciousness may find its inspiration from an unlikely source: literature.

### Morel's invention as inspiration for an integrative description of consciousness

**Figure 1:**
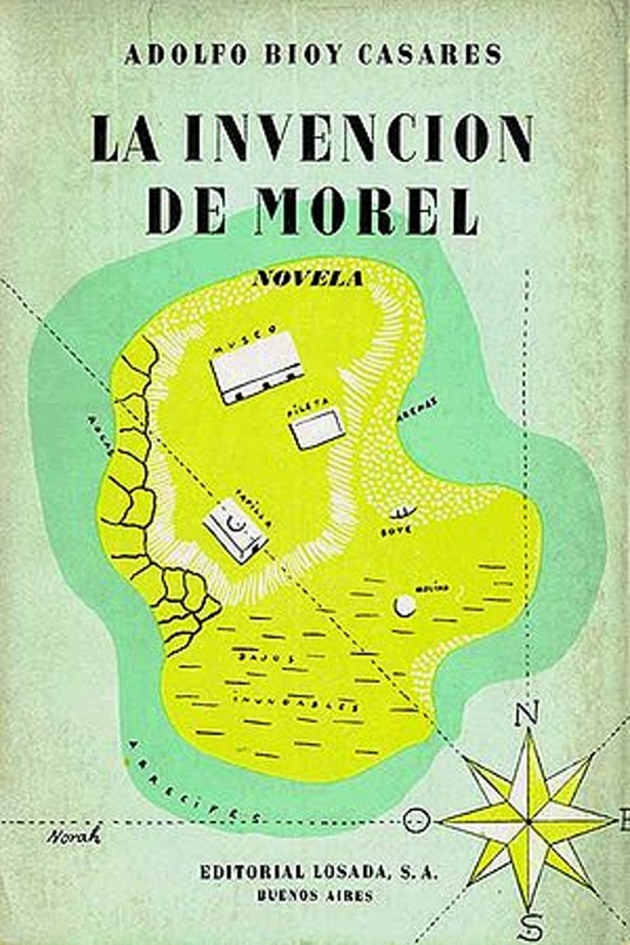
**The first Spanish edition of “The Invention of Morel”, depicting the map of the island where the scientist Morel would have created and used his innovating machine.** Copyright: Editorial Losada S. A., Buenos Aires, 1940, Cover by: Norah Borges.

Adolfo Bioy Casares (1914–1999) was an Argentinian author, who was born and lived in Buenos Aires. He is most famous for his early fantasy novel “The Invention of Morel,” which was first published in 1940 (Figure 1) and which blends elements of science fiction, romance, and philosophy. Most of the story describes a criminal's thoughts, fears, reactions, and puzzlement over discovering the inhabitants of an isolated island of the Pacific, on which he arrives as a fugitive. During the day, these persons act in a stereotyped manner, repeating the same behaviors and having the same discussions. During the night, they seem to disappear from the island. The protagonist, who is constantly afraid they will discover him and turn him in to the authorities, decides to approach a woman (Faustine), to whom he feels attracted. It turns out that this woman, as well as other inhabitants, totally ignore his presence. Other strange observations include the presence of two suns in the sky and the discovery of identical copies of dead fish he found on his day of arrival. Slowly he starts to question himself as to whether his perception is reliable and if he actually dreams or hallucinates (Casares, 1940, p. 44, 52). While the readers of this novel may also come up with all sort of theories about what is happening on the island, we finally find out the truth: Morel, who is the scientist on this island, invented a machine, which could capture and store “*waves*” and “*vibrations*” of all human senses (visual, auditory, olfactory, somatosensory, and gustatory). Images and frames of a human being could also be captured, recorded, and projected by motion picture and mirror technology (in the absence of relevant scientific evidence, Casares gives little rational explanation for a technique which would be later referred to as holography). When the sensory waves were synchronized with the images projected in space, the person and his surroundings in their totality were reproduced exactly as they were in their initial form. These projections had weight, depth, height, and appeared as real persons, engaging all five senses. The machine projected on to the whole island motion images, which had been recorded during the period of 1 week, and did this in a looping mode that would last forever. Thus, the protagonist learns that he is surrounded by reproduced three-dimensional images of persons who existed only in the past. After a short period of repulsion about this “fake reality,” the protagonist accepts its existence as something better than his own. The novel ends when the fugitive learns how to operate the machine and inserts himself into the recording so it looks as if he and Faustine are in love.

In “The Invention of Morel,” Casares examines the fundamental philosophical problems of perception and consciousness. Influenced by George Berkeley's *subjective idealism* (Berkeley, 1713), a doctrine that supports the theory that only mind and mental experiences exist and that physical objects do not exist except as perceptual phenomena, the author questions whether reality is an exclusive creation of the mind and if human is able to perceive the world accurately through his senses. According to Casares, human perception will always be subjective; he symbolically represents this claim with a machine, which reproduced perceptual reality and modulated the subject's (fugitive) perceptual experience, emotions, and thoughts. For Morel, the ultimate use of his machine would be the “*eternity of consciousness*”: by repeating consecutively the moments of 1 day or 1 week, “*we are powerless to escape from the consciousness we had in each one of those moments and we shall have no memories other than those we had in the corresponding moment of the eternal record. The future, left behind many times, will thus maintain its attributes forever*” (Casares, 1940, p. 76).

The scientist considered that “*we have no valid reason to deny consciousness to the persons created by his machinery*” (Casares, 1940, p. 71). The protagonist of “Invention of Morel,” becoming aware of the forces that this machine can have, proposes that it should be enhanced with the capacity to describe the thoughts, emotions, and other brain states of a subject any distance away from him/her (Casares, 1940, p. 82). This enhanced machine would offer the ability to describe consciousness, as “*one's thoughts and feelings during life will be like an alphabet with which the image will continue to comprehend all experience*” (Casares, 1940, p. 82). Although Morel's machine was not such a brain reading machine, and Casares' idea may have seemed naive and totally fictive until some years ago, astonishing progress in several facets of cognitive neuroscience, functional neuroimaging, and computational neuroscience, now permit us to wonder if decoding perceptual reality and mental states from brain activity is possible.

So, according to Casares' novel, a “consciousness device” should satisfy two simultaneous conditions:

1. Multimodal perception binding [“*when all senses are synchronized, the soul emerges*” (Casares, 1940, p. 71)]. This condition is in accordance with many philosophers and neuroscientists, who consider that consciousness is characterized by the apparent unity of perceptual experience despite multiple sense modalities (Llinas et al., 1998; Searle, 2000; Llinas, 2008). We will here see that virtual reality technologies can enhance or alter aspects of unity and subjectivity (e.g., self-processing, bodily self-consciousness) and thus inform us about these aspects of consciousness (Blanke, 2012; Tajadura-Jimenez et al., 2012).
2. Brain reading of qualitative conscious experiences. Brain reading is now considered as a very promising novel approach to the study of specific conscious states (Weil and Rees, 2010). Apart from its utility in non-communicating subjects (e.g., locked-in patients), this method may be also capable of offering a more objective measure of conscious states than traditional fMRI or behavioral methods, by bypassing biases of the subject-examiner interaction (e.g., suggestibility).

The departure point of this article is to draw analogies between a literary device (Morel's invention) and current progress in virtual reality (chapter “Reinventing Morel's Machine I: Virtual Reality Component”) and brain reading (chapter “Reinventing Morel's Machine II: Brain Reading Component”). These technologies will be described briefly, and will be proposed as necessary and complementary tools to simultaneously gather first-person (virtual reality) and third-person (brain reading) data (chapter “Combining Virtual Reality and Brain Reading Technologies in Order to Describe Consciousness”). Bioy Casares proposes such an integrated model of consciousness, even if only in the form of philosophical idea and not of scientific expertise. Later in the article, we will wonder if the multimodal, simulating, and integrative capacities of the dreaming brain can reliably inform us about consciousness (chapter “The Dreaming Brain as an ‘Endogenous’ Morel's Machine and its Relation to Consciousness”). Both the literary and dreaming machines, and their integrative characteristics, could inspire cognitive scientists to create new experiments and similar combined (virtual reality/decoding) devices.

## Reinventing morel's machine I: virtual reality component

Although Morel's invention lacks scientific value *per se*, the idea was innovating. Current virtual reality technologies can create, modulate, and alter perceptual and physical reality, similarly to Morel's machine (Lenggenhager et al., 2007; Suzuki et al., 2012). Substitutional reality (SR) refers to reproducing, modulating, or substituting live reality without noticing the change. This is possible with the aid of virtual and mixed reality techniques (VR/MR), like a head-mounted display (HMD), a control computer, and a panoramic video camera (SR system) (Suzuki et al., 2012). The SR system can ensure natural visuo-motor coupling of real-time scenes and previously recorded scenes, so that participants wearing the HMD can experience both kind of scenes within a continuum, without noticing a reality gap. These experiences can be inconsistent, like for example encountering themselves (Doppelgänger scene). They can even experience identical episodes repeatedly (e.g., conversations, movements) in an eternal *déjà-vu* situation. Very interestingly, these experiences share an astonishing resemblance to Casares' world. Pre-recorded scenes by a panoramic camera have been used in both SR system and Morel's machine, making Casares' novel both prophetic and scientifically innovative, considering the year of its first publication (1940). Other methods used for SR are mixed reality (MR) system (Costanza et al., 2009) and diminished reality (DR) system (Herling and Broll, 2010), which allow interactions of a person with a fictive environment, using more than one sense in an integrative way. Other scientists, using virtual reality and multisensory conflict, succeeded in creating an illusion where healthy participants experienced a virtual body (*avatar*) as if it were their own (Lenggenhager et al., 2007). Furthermore, the participants said that the sensorimotor experiences of the avatars felt like their own. Importantly, most of the subjects using the aforementioned virtual technologies do not doubt their false perception (Lenggenhager et al., 2007; Slater, 2009; Suzuki et al., 2012), a reaction similar to the dreaming state (chapter “The Dreaming Brain as an ‘Endogenous’ Morel's Machine and its Relation to Consciousness”).

According to Damasio “*consciousness occurs when we can generate, automatically, the sense that a given stimulus is being perceived in a personal perspective; the sense that the stimulus is ‘owned’ by the organism involved in the perceiving; and, last but not least, the sense that the organism can act on the stimulus (or fail to do so)*” (Damasio, 1998). These criteria can be fulfilled in a virtual environment (Sanchez-Vives and Slater, 2005), where many aspects of subjective states (self-identification, interoceptive sensitivity, bodily, and emotional experiences) and self-perception (multisensory binding) can be controlled experimentally. More specifically, by manipulating several parameters, such as graphics frame-rate, sound, haptics, virtual body representation, and movement, we can modulate the qualitative and subjective attributes of a virtual experience. Inside such an environment, enhanced verbal reports (Witmer and Singer, 1998), or behavioral measures, such as the postural sway (Freeman et al., 2000) and the resolution of conflicting multi-sensory cues (Maravita et al., 2003), or physiological measures, such as the autonomic bodily responses (e.g., heart rate, skin conductance), can be used to measure subjective experiences (Insko, 2003). Therefore, and in accordance with others (Lenggenhager et al., 2007; Slater et al., 2010; Tajadura-Jimenez et al., 2012), I propose that virtual reality technologies or similar techniques (e.g., mirrors, illusions) can be useful in the description of first-person data. Their use may bring us a step closer to studying the question of *qualia*, that is, our subjective, conscious experience of things, which has specific properties and neural underpinnings (Llinas, 2002), and which has been largely neglected by experimental studies. As Antti Revonsuo stated, “*The virtual reality-community has done a valuable service to consciousness research, because what they have come up with are terms that capture the realization of subjective phenomenal organization from the first-person's point of view*” (Revonsuo, 1999, p. 92).

## Reinventing morel's machine II: brain reading component

By modulating subjective experiences, virtual reality can offer promising scientific investigation of the ontological subjectivity. However, an objective measure of this experience will be only possible by studying its neurophysiological correlates. This can be accurately achieved by brain reading. Brain reading refers to the decoding of perceptual or mental conscious states by brain activity alone. The modern methods of “brain reading” are essentially two: invasive techniques, which include electrode implantation in the brain (Kennedy and Bakay, 1998), and non-invasive techniques, which refer to conventional and multivariate neuroimaging approaches (Haynes and Rees, 2006). They permit decoding of mental states with high accuracy and reasonable temporal resolution. Multivariate pattern analysis (MVPA) is the most common technique used in brain reading studies (Weil and Rees, 2010).

In this article I will only briefly describe the most recent and important findings on brain reading of conscious states, as a more exhaustive analysis is not the objective of this article, and it has been already done elsewhere (Haynes and Rees, 2006; Tong and Pratte, 2012).

### Decoding perceptual reality from brain activity

**Figure 2:**
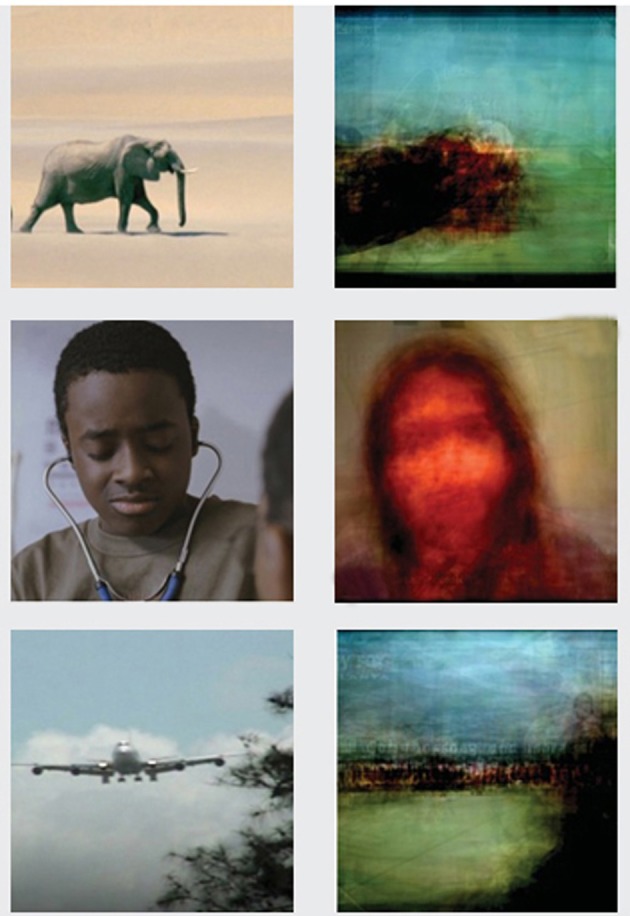
**On the left: visual experience from natural movies.** On the right: reconstruction of these images by decoding brain activity. Adapted from movie S1 published in Current Biology, 21, Nishimoto, S., Vu, A. T., Naselaris, T., Benjamini, Y., Yu, B., and Gallant, J. L. “Reconstructing visual experiences from brain activity evoked by natural movies.” Supplementary Information. Copyright: Elsevier 2011. Figure approved by corresponding author Jack Gallant.

Morel's visual “waves and vibrations” would correspond to visual experiences reconstructed from brain activity (BOLD signals or EEG). Such a reconstruction was recently described by several groups (Kamitani and Tong, 2006; Kay et al., 2008; Miyawaki et al., 2008; Nishimoto et al., 2011). Tellingly, Miyawaki et al. reconstructed visual images from fMRI signals of the human visual cortex on a single trial basis. A large variety of images (2^100^ possible images) were accurately reconstructed without any prior information about the image. On the other hand, the Gallant group (Naselaris et al., 2009; Nishimoto et al., 2011) presented a motion-energy model of visual reconstruction. They described a Bayesian decoder using fMRI signals from early and anterior visual areas in order to reconstruct complex natural images. They extended this decoder so that it could model brain activity elicited by dynamic stimuli such as natural movies in the occipitotemporal visual cortex of human subjects (Figure 2). Based on these models, future studies could further improve reconstructed image quality by increasing the number of video images shown during the encoding and by integrating new knowledge about the neural representation of visual images.

Reconstructing auditory attributes, like hearing spoken language was also described (Formisano et al., 2008; Raizada et al., 2010; Abrams et al., 2011). Most recently, Pasley et al. (2012) used intracranial recordings from the auditory cortex of patients while they listened to a sequence of different words. By recording the neural responses from the superior temporal gyrus using spectrogram and modulation-based auditory representations, they succeeded in accurately reconstructing syllable rate, syllable onsets, and offsets, and individual words, directly from brain activity.

Patterns of activity in the human posterior piriform cortex were found to predict accurately the perceptual ratings of odor quality (Howard et al., 2009). In the future, similar (pattern-based) techniques could be used to predict distinct olfactive experiences by recording odor-evoked response potentials from olfactory receptor neurons in response to distinct olfactory stimuli with an electro-olfactogram (Lapid and Hummel, 2013).

### Decoding and predicting mental states from brain activity

#### Decoding mental imagery and memory

An important question that confronts current research in neuroscience is whether we can predict mental states, like emotions, memories, and thoughts based on the measurement of brain activity alone (a question that the protagonist of “The Invention of Morel” also had). Indeed, individual introspective mental events, like imagination of a face or a house, can be tracked from brain activity in highly specialized regions of the ventral visual pathway, like the fusiform face area or the parahippocampal place area (O'Craven and Kanwisher, 2000). Decoding of object representation (e.g., chairs, shoes, animals) from response patterns in object-selective regions of the temporal lobe is also possible due to pattern-based analysis or support vector machines (Haxby et al., 2001; Cox and Savoy, 2003). The orientation, direction of motion, and perceived color of a visual stimulus can also be predicted by decoding patterns of signals from the early visual cortex (Kamitani and Tong, 2005).

Recently, decoding long-term episodic memories while they are being recalled by the participants was also achieved. After training pattern classifiers to discriminate between certain categories (famous places, persons, or common objects), that were shown to participants under fMRI, a correct prediction of category was achieved upon subsequent retrievals (Polyn et al., 2005). A similar prediction of other complex episodic memories (e.g., movie recall or spatial memories) was also achieved by activity in the hippocampus (Chadwick et al., 2010; Morgan et al., 2011). Other fMRI studies decoded semantic memory (Naselaris et al., 2009) or predicted brain responses to novel nouns associated with verbs of known semantic attributes (e.g., see, hear, eat, or touch) (Mitchell et al., 2008).

#### Predicting emotional states and decision choices

Emotions such as happiness, sadness, fear, and anger activate networks of several cortical and subcortical brain regions such as insula, amygdala, hippocampus, thalamus, medial and lateral prefrontal cortex, orbitofrontal cortex, and anterior cingulate cortex (Phan et al., 2002). Could we accurately predict these emotional states only from brain activity (fMRI signals)? Strong responses in an extended limbic circuit (amygdala, insula, transverse temporal gyrus, temporal operculum, planum temporale, and inferior frontal gyrus) after an emotional stimulus (mother's voice) were found in the Minimally Conscious State (Bekinschtein et al., 2004). More recently, an online support vector machine was able to reliably predict and decode three discrete emotional states (happiness, disgust, and sadness) from brain activity while individuals performed emotion imagery (Sitaram et al., 2011). Involvement of the frontal cortex, anterior cingulate cortex, and insula in emotion imagery was also revealed. In another study, resting brain metabolism in the amygdala, the dorsal anterior cingulate cortex, and the ventromedial prefrontal cortex predicted responses during fear extinction and extinction recall (Linnman et al., 2012). Future experiments should address predictability of other emotional states too, as well as construction of subject-independent brain state classifiers, as the aforementioned studies require collection of initial data for classifier training.

Similarly, using classifier learning from a simple reward-based decision-making task and a multivariate analysis approach, an accurate prediction of subsequent behavioral choices was achieved (Hampton and O'Doherty, 2007). Activation of three regions of interest out of nine (anterior cingulate cortex, medial prefrontal cortex, and ventral striatum) was sufficient for the prediction. A similar study design for purchasing decisions has shown that activity of the nucleus accumbens and medial prefrontal cortex can correctly predict subsequent purchases (Knutson et al., 2007).

## Combining virtual reality and brain reading technologies in order to describe conscious states

Bringing to mind Casares hypothesis, I propose here that virtual reality and brain reading can inform us conjointly about consciousness. Virtual reality will provide scientists with the ability to experimentally examine the component of subjectivity and unity of a subject's conscious state, whereas brain reading will provide us with the objective measure of the same state.

Theoretically, an on-line decoding “consciousness device,” could consist of a virtual environment system, easy to use for daily operation, such as the SR system [as described in Suzuki et al. (2012)] combined with an fMRI decoder, according to what conscious state we want to examine (e.g., visual experiences, mental imagery, emotional experience). By means of the SR system, the examiner could induce specific and targeted subjective conscious states in the subject and use the measures of subjective experience exposed previously (enhanced verbal report, behavioral, physiological measures) (see chapter “Reinventing Morel's Machine I: Virtual Reality Component”); the fMRI decoder could then provide an objective measure of this conscious state. Importantly, the subject can be informed about the objective measure of his/her experience (brain processes), what could further improve the reliability of his/her verbal reports (metacognitive properties). Future experiments could be based on this model, although improvement in temporal and spatial resolution of current neuroimaging techniques is certainly needed.

It should also be noted that, to this date, decoding information from several simultaneously occurring modalities (e.g., vision and attention, audition and memory) is not possible, as current methodologies cannot overcome the potential spatial and neurobiological superposition of these states. Therefore, improvement of statistical pattern recognition algorithms is required.

## The dreaming brain as an “endogenous” Morel's machine and its relation to consciousness

Based on neuroscientific, philosophical, and artificial intelligence models, we have until now assumed that a better description and representation of consciousness should include multimodal binding and flexible access to subjectivity and to qualitativeness of conscious states. Virtual reality and brain reading could offer us such integration. A “consciousness” device being still inapplicable due to restrictions of current techniques, studying internal states with similar multimodal perceptual integration and simulation of subjective, qualitative experiences, such as dreaming, could further elucidate our understanding of conscious states. The question that is then posed is: can dreaming reliably inform us about consciousness?

### Dreaming can inform us about consciousness

Studying dream states can potentially contribute to the investigation of some of the neurobiological, representational, and functional aspects of self-consciousness.

At first glance, the dreaming brain could be considered as an ideal “endogenous” Morel's machine. It integrates successfully (Hong et al., 2009) the sensorimotor plus cognitive and emotional modalities described previously in the article, into a subjective experience. The most prevalent sensory modality in dreams is vision (100%) and audition (40–60%), while movements and tactile sensations (15–30%) or smell and taste (less than 1%) are less frequent (Strauch et al., 1996). Thalamocortical signaling during sleep lowers the thresholds of associative areas so that they produce perceptual and cognitive experience, even in the absence of external sensory input. More specifically, activation of the visual association cortex and parieto-temporo-occipital (PTO) junction appear to be responsible for visual imagery and spatial cognition during dreaming (Braun et al., 1997; Nofzinger et al., 2002). Activation in auditory temporal cortices and motor circuits (including the cerebellum and basal ganglia) (Braun et al., 1997) is consistent with auditory elements and fictive motor actions respectively. Moreover, activation of motivational, memory, and emotional circuits (Perogamvros and Schwartz, 2012) may reflect active reward processing, memory consolidation, and emotion regulation processes during sleep. Interestingly, memory processing seems to initially precede and initiate visual imagery in dreaming (Ji and Wilson, 2007), in agreement with theories proposing that sleep-dependent memory-reward processing is responsible for dream generation (Freud, 1900; Solms, 2000; Wamsley and Stickgold, 2010; Perogamvros and Schwartz, 2012). We should note that the synchronous activity in the thalamocortical system, considered as the *core* of consciousness (Llinas and Ribary, 2001), is present in both wakefulness and sleep (Llinas and Ribary, 1993). Therefore, intrinsic activity in this system during dreaming could inform us about waking conscious states.

Dreams also share common features with virtual reality, as they are characterized by misinterpretation of the perceptual input (the dreamer considers the sensory stimuli as incoming), and by perceptual distortions, such as alterations in spatio-temporal integration, bizarreness, and out-of-body experiences (*heautoscopy*). Most of these features may relate to changes in regional brain activity and in functional connectivity between brain regions (Maquet et al., 2005; Massimini et al., 2010). The ability of the brain to accept a fictional reality it creates as an actual one (adhesion), and thus alter the state of its own perception, is related to activations of the left ventral inferior frontal gyrus and left posterior superior temporal sulcus (Metz-Lutz et al., 2010). Interestingly enough, both regions display increased activity during sleep compared to wakefulness (Braun et al., 1997; Dehaene-Lambertz et al., 2002; Kaufmann et al., 2006). Perceptual experience in dreams, contrary to waking experience, is a genuine representation of *subjective idealism* (see chapter “Morel's Invention as Inspiration of an Integrative Description of Consciousness”), because all sensory stimuli exist solely as perceptual brain products. Therefore, by studying perceptual and cognitive subjectivity as represented in dreams, dream research could contribute to the theoretical understanding of first-person data.

Finally, it has been proposed that off-line emotional maturation takes place during Rapid eye movement (REM) sleep since as early as the 13th week of gestation (Kurjak et al., 2005; Dondi et al., 2007) and that quiet sleep of human neonates contributes to the formation of cortical connections required for sensorimotor coordination and body representation (Milh et al., 2007). Overall, sleep seems necessary for the genesis of consciousness and self-awareness via the interaction of genetic instructions with off-line sleep-dependent perceptual experiences (Hobson, 2009; Perogamvros, 2012). In addition, dream consciousness seems important for the development and maintenance of waking consciousness and cognition, by contributing to sensorimotor integration, virtual reality simulation, and performance improvement in an off-line mode (Revonsuo, 1995; Perogamvros and Schwartz, 2012).

### Dreaming is not always reliable for studying consciousness

Dreaming offers a unique condition of internal information processing, which is functionally isolated from the external world. Indeed, dream consciousness, which may arise as ascending activation of attractor networks of visual and multimodal association areas, is, at least partially (Arzi et al., 2012), disconnected from the external environment. This is due to domination of internally oriented cortical networks, like the brain default network (Domhoff, 2011), over externally oriented cortical networks. This disconnection of the internal dreaming state from the external environment explains why, up until now, the sole way of studying dreams is to base the examination on subjective parameters: what the subject remembers of his/her dream after being awakened. Consequently, decoding a dream state with lack of objective information about the encoded state, reduces significantly the accurate assessment of the examined state.

**Figure 3:**
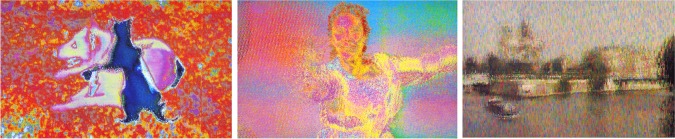
**Image reconstructions from dreams using a dream decoder in the Wim Wenders film “Until the end of the world” (1991)**. “Dog and cat,” “Woman running,” and “Paris,” Wim Wenders 1991, Electronic Paintings from “Until the end of the World” by Wim Wenders, 1994 (Director's Cut). Copyright: Wim Wenders Foundation, Düsseldorf.

Indeed, reconstructing dream reality with a dream decoder and thus being able to visualize dreaming experience remains a staple of science fiction stories. Theoretically, internally generated signals from the PTO junction could be reconstructed similarly to the reproduction of external static or dynamic visual stimuli (as described in “Decoding Perceptual Reality from Brain Activity”). However, considering that the dreaming experience is multimodal, an ideal dream decoder should be based not only on sensory decoding models (Nishimoto et al., 2011; Pasley et al., 2012) (see section “Decoding Perceptual Reality from Brain Activity”), but also on semantic (Schwartz, 2004; Mitchell et al., 2008; Andrews et al., 2009), episodic (Polyn et al., 2005), emotional (Sitaram et al., 2011), and motor (Dehaene et al., 1998) models. Importantly, this multimodal decoder would need sophisticated pattern-classification algorithms, due to increased novelty in dreamed representations (Perogamvros and Schwartz, 2012). Interestingly, the image reconstructions of natural movies of Nishimoto et al.'s decoder (Nishimoto et al., 2011) (Figure 2) share a striking similarity to the dream images reproduced by a visual dream decoder in the Wim Wenders' film “Until the End of the World” (1991) (Figure 3).

Apart from technical difficulties in the evaluation of decoding accuracy of dreaming, there are several phenomenological reasons for which dreaming cannot always offer a reliable description of consciousness. These are described in the dream argument and the functional dissociation argument.

The dream argument (Zhuàngzi, 369 BCE; Plato, 360 BCE; Aristotle, 350 BCE; Descartes, 1641) postulates that dreaming and waking life experiences are not easily distinguishable, because they are both based on a potentially faulty perceptual system. This skeptical hypothesis claims that the sleeping mind is not a reliable mechanism for attempting to differentiate reality from illusion. What may actually separate waking from dream consciousness is their different degree of coherence (waking experience > dreaming experience) and the assumption that we can more easily explain dream content by means of waking-life experiences than the reverse. However, the distinction between the two states can only be approximate, as recent experiments are in support of the dream argument (Mazzoni and Loftus, 1996).

The functional dissociation argument described here considers that dream consciousness and waking consciousness may not be functionally related. Although there is evidence that past and current waking concerns influence dream content (Cartwright et al., 2006), dream consciousness is largely characterized by novelty and creativity. Indeed, while a large proportion of dream elements comes from recent memory (Schwartz, 2003), integrated life episodes are incorporated in no more than 1–2% of dreams reports (Fosse et al., 2003). Dreams are usually novel constructions and rarely reproductions of past events (Meier, 1993). This leads inevitably to the question: is the dreaming life deterministically secondary to our waking life? In other words, is sleep the “servant” of waking life and do the activations of brain networks during dreaming reflect a function sleep serves for waking life or for itself? Could it be that the awakened *avatar* in our dreams has an *independent Self* and leads a “second life,” which is independent of our wakefulness? Current neuroimaging studies compare and interpret the activation/deactivation patterns in sleep in relation to wakefulness, assuming that the latter defines a principal state of reference. However, activated emotional and reward networks as well as deactivated reality-monitoring structures in sleep could define a *protoconscious state* (Hobson, 2009), whose existence and roles may be completely independent of and distinct from the waking conscious state. This statement may be partially supported by evidence that congenitally blind people can have visual dreams (Lopes Da Silva, 2003). Similar findings have been demonstrated for congenitally paraplegic and deaf-mute persons, who have dreams with modalities not experienced during waking life (Voss et al., 2011). Therefore, a possible functional dissociation between the waking Self and the dreaming Self [*protoself* according to Hobson (2009)], should be addressed in future studies. As J. L. Borges imagined in 1940 in his story “Tlon, Uqbar, Orbis Tertius” (Borges, 1944):

“while we are asleep here, we are awake somewhere else, and every man is thus two men.”

## Conclusions

In this article, a literary source (The Invention of Morel) has been the inspiration for an integrative model of consciousness. The combined binding/brain reading approach of Casares would sufficiently describe the main attributes of consciousness: unity, subjectivity, and qualitativeness (Searle, 2000). With current technologies, this approach would correspond to a combined virtual reality/multimodal decoder, which could manipulate subjective conscious states (virtual reality component) and decode them (brain reading component). This approach could simultaneously gather first-person data with third-person data.

The association of virtual reality technologies and decoders seems necessary, as brain reading techniques alone offer insufficient description of consciousness. Indeed, brain reading informs us accurately and with reasonable temporal resolution about the neurophysiological correlates of conscious states, but not about subjectivity. On the other hand, virtual reality technologies, already used to explore self-consciousness (Sanchez-Vives and Slater, 2005; Lenggenhager et al., 2007), offer a promising and sophisticated methodology for investigation of subjective experience.

Although some preliminary results demonstrate that integration of first-person data and third-person data may be possible with current methodologies (Ijsselsteijn et al., 2001; Lutz et al., 2002; Dehaene et al., 2003), restrictions in temporal and spatial resolution of neuroimaging techniques render studies investigating associations between subjective states and objective measures, difficult to perform. Future experiments should address these issues.

Faced with these difficulties, studying an internal state incorporating multimodal integration and reality simulation, such as dreaming, could further elucidate the nature of conscious states. Although technical difficulties (to date, verbal reports are the only method of studying dreams) and questions about phenomenology (the dream argument and the functional dissociation argument), render dreaming a not always reliable model for consciousness, it may still answer some crucial questions of neurobiological, representational, and functional aspects of self-consciousness, such as perceptual binding, sensorimotor integration, and self-identification.

### Conflict of interest statement

The author declares that the research was conducted in the absence of any commercial or financial relationships that could be construed as a potential conflict of interest.

## Abbreviations

BOLD: Blood-Oxygen-Level-Dependent
DR: Diminished Reality
EEG: Electroencephalography
fMRI: functional Magnetic Resonance Imaging
HMD: Head-Mounted Display
MVPA: Multivariate Pattern Analysis
PTO: Parieto-Temporo-Occipital
REM sleep: Rapid Eye Movement sleep
SR: Substitutional Reality
VM/MR: Virtual and Mixed Reality.
